# Effects of a Post-Traumatic Growth Program on Young Korean Breast Cancer Survivors

**DOI:** 10.3390/healthcare11010140

**Published:** 2023-01-01

**Authors:** Ka Ryeong Bae, Wi-Young So, Seyong Jang

**Affiliations:** 1 Samsung Advanced Institute of Health Sciences & Technology (SAIHST), Sungkyunkwan University, Seoul 06355, Republic of Korea; 2 Sports Medicine Major, College of Humanities and Arts, Korea National University of Transportation, Chungju-si 27469, Republic of Korea; 3 Department of Taekwondo, College of Arts and Physical Education, Gachon University, Seongnam 13120, Republic of Korea

**Keywords:** breast neoplasms, cancer survivors, post-traumatic growth, return to work

## Abstract

Background: This study aimed to enhance post-traumatic growth in young breast cancer patients by providing them with self-disclosure and social support resources, to reduce intrusive rumination and increase deliberate rumination. This study was conducted at a university-based cancer center in Seoul, South Korea. The study included a four-week group-based psychoeducational program, which supported the post-traumatic personal growth of breast cancer patients. The primary outcome was increased post-traumatic growth, and the secondary outcomes were rumination, distress, and traumatic perception. Methods: The study included 38 young breast cancer patients, with a mean age of 42.21 years and mean time since diagnosis of 13.84 months. Results: Results of the study revealed statistically significant improvements in total post-traumatic growth and all subdomains, after the intervention (*p* < 0.001) and one month later (*p* < 0.001), as compared to the baseline. A significant improvement was also observed in deliberate rumination after the intervention (*p* = 0.038). Furthermore, the patients had a statistically significant decrease in anxiety, after the intervention (*p* = 0.035) and one month later (*p* = 0.005), as compared to the baseline. Conclusions: Overall, the program encourages activities that promote post-traumatic growth, enabling young breast cancer patients to return to their normal lives. Specifically, the growth allows patients to return to work and maintain a healthy body and mind. The results of this study indicate that this program can be used as an effective psychological intervention for young breast cancer patients.

## 1. Introduction

Compared to other countries, South Korea has the highest global rate of breast cancer among women in their 40s. However, breast cancer has a high five-year survival rate of 93.6% [1], due to early detection and the standardized treatment methods in South Korea. As such, breast cancer patients require continued healthcare after the cancer has been treated. In addition to the process of diagnosis and treatment, breast cancer patients experience both physical symptoms (pain, fatigue, and lymphedema) and emotional symptoms (depression, body dysmorphia, anxiety, and fear of recurrence) [2]. As a result, patients with breast cancer are at an increased risk of psychiatric disorders, including post-traumatic stress disorder [3].

Notwithstanding the rising survival rate of cancer patients, cancer remains a serious life threat. It is often perceived as a “suddenly encountered traumatic event”, characterized by uncertainty about the future and changes in social roles and relationships [4,5,6]. Although cancer patients face negative experiences after being diagnosed with cancer, they may also experience positive effects, such as promoting personal growth by focusing on the spiritual benefits that accompany emotional distress, discovering a new meaning or purpose in life after cancer [7]. Patients may also experience positive psychological changes through discovering internal growth, shifting perceptions about life, and building rich interpersonal relationships [8]. Such positive psychological changes are referred to as post-traumatic growth (PTG) [9]. PTG not only promotes psychological adaptation and improves quality of life in cancer patients but also facilitates health-promoting behaviors and increases immunity and survival rates [10,11,12,13]. Therefore, it is important to develop intervention strategies that promote PTG in patients with cancer, so that they may continue to lead healthy lives. However, studies that focus on increasing PTG levels are limited. As a result, strategies are needed to help cancer patients promote PTG and further reduce psychological distress, thereby improving their quality of life [14].

According to Li et al. [15], psychosocial interventions are the most effective in improving PTG in cancer patients. Specifically, mindfulness-based interventions have been shown to be effective for breast cancer patients [15]. However, it is difficult to provide extended psychosocial interventions for cancer patients who are undergoing or have finished treatment. As few clinical psychologists are present in hospitals or communities, it is necessary to develop an effective PTG program that can be implemented by nurses. In addition, many PTG intervention studies lack consistent results, either because PTG is explained only as a positive outcome of the intervention or because there is no clear explanation of the underlying process [16,17]. Based on the theory posited by Tedeschi and Calhoun [9], this study developed a PTG promotion program (Join with us for Unique Worker and Move on Posttraumatic Growth; JUMP program) to be implemented by nurses. We aimed to evaluate its effectiveness in this study. The results will help young breast cancer patients return to their daily lives, including returning to work and maintaining a healthy body and mind through PTG.

## 2. Materials and Methods

### 2.1. Participants

This study was conducted from November 2016 to January 2017 at a university-based hospital cancer center in Seoul, South Korea. The inclusion criteria were as follows: adults aged 19–50 years who had received or completed active cancer treatment after being diagnosed with breast cancer and who could communicate, understand, and respond to questionnaires in Korean. The exclusion criteria were as follows: patients with mental disorders or other severe complications, patients currently participating in another intervention program, and patients who could not continue participating in the education program.

The announcement of program participant recruitment was posted in the appropriate online community. We created a mobile website to provide information about and advice on the program. Those who wished to participate in the study voluntarily applied via phone, text message, or email. The researcher checked the physical and mental status of the participants and then explained the purpose and content of the study. Candidates who provided informed consent were enrolled in the study. The program required small groups of about six people per session. Given a 20% withdrawal rate, up to eight people were able to participate in a session. A total of 38 participants were recruited for three months, and all completed the intervention.

The average age of the 38 participants was 42.21 years. Among the participants, 26 (68.4%) were in their 40s, more than two times the number in their 30s. Overall, 30 (78.9%) participants had a spouse, 26 (68.4%) belonged to a religion, and 36 (94.8%) had a university degree or higher. Thirty-three (86.8%) participants had a monthly household income of ≥ USD 3000. Additionally, Stage I cancer diagnoses were most common among participants (34.2%). The average time since diagnosis was 13.84 months, with 21 participants (55.3%) having been diagnosed within six months to a year of the study. Of the 37 (97.4%) patients who underwent breast surgery, 22 (57.9%) had breast-conserving surgery. Twenty-nine participants (76.3%) received chemotherapy, and twenty-eight (73.7%) received radiation therapy. Twenty-one (73.7%) participants received anti-hormone therapy. Additionally, 21 (55.3%) participants were clerks. The average working period was 127.29 months, and 26 (68.4%) participants were on a leave of absence (Table 1).

### 2.2. PTG Promoting Program (JUMP Program)

**Figure 1:**
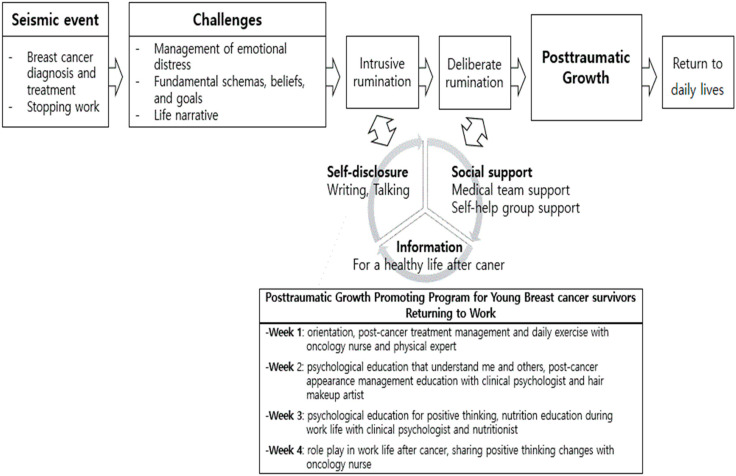
Contents of post-traumatic grow program on young breast cancer patients.

Based on the PTG model, the most important components of the program were increasing self-disclosure and social support. By enhancing these aspects of patients’ lives, the program aimed to facilitate deliberate rumination, thus promoting PTG (Figure 1).

They following needs were identified for patients participating in the PTG program: measures for patients to relieve their difficulties in undergoing treatment and maintaining a job, professional insight into healthy diet and exercise, esthetic management and counseling, and support groups for patients to express their feelings with peers. We determined that the intervention program should help patients understand the meaning of breast cancer, learn methods to cope with various situations, and plan a new life after cancer. These skills would allow patients to minimize the challenges of cancer and to achieve internal and external growth. The preliminary program was developed based on the findings obtained during the design stage, and the content validity was verified by an expert panel. Additionally, the structure, format, and content of the program were verified by seven experts. The program was then modified to fit patient needs. As young female patients often have difficulty finding childcare for weekday activities, the study ran both weekday and weekend classes simultaneously.

Each session of the program was divided between self-disclosure and social support resources. Additionally, post-cancer treatment management, exercise, psychology, nutrition, and esthetic management were divided into four sessions as part of a two-hour program. The program also provided group and individual interventions. Group intervention was provided in every session; during this time, the participants attended lectures at the hospital’s lecture hall or participated in discussions or role-playing activities. Individual interventions involved counseling via social networking sites and a mobile community. Through this platform, participants were able to express themselves in writing, drawings, and photographs at any time.

### 2.3. Measurements

Participants completed self-report questionnaires three times during the study period: before the program (T1), immediately after program completion (T2), and one month after program completion (T3). Measurement of PTG in breast cancer patients was developed by Tedeschi and Calhoun [9] and Song et al. [18], using the Posttraumatic Growth Index (PTGI) validated in Korean. The PTGI has 21 questions, and each question can be answered using a six-point Likert scale. This tool consists of five subscales: relation to others, new possibilities, personal strengths, spiritual changes, and appreciation of life. Total scores range from 0 to 105, with higher scores reflecting positive post-traumatic changes.

Intrusive and deliberate rumination are cognitive processes that occur in patients who are diagnosed with breast cancer and undergoing treatment. These aspects were measured in participants using the Event-Related Rumination Inventory in a Korean Population (K-ERRI), which was originally developed by Cann et al. [19] and adapted for use in Korea by Ahn et al. [20]. The K-ERRI comprises 20 items, with 10 items for both intrusive and deliberate rumination. The subscore of each rumination type ranges from 0 to 30, with a higher score indicating greater reliance on the corresponding type of rumination.

After experiencing breast cancer diagnosis and treatment, patients often suffer psychological pain as a result of failing to achieve growth, and they may even continue to suffer even after growing from the experience. This distress was measured using both anxiety and depression. The Korean standardized version [21] of the Hospital Anxiety and Depression Scale (HADS) was developed by Zigmond and Snaith [22]. The HADS comprises 14 items, with 7 odd-number subscales (HADS-A) and 7 even-number subscales (HADS-D). The total score ranges from 0 to 21, with higher scores indicating higher levels of anxiety and depression.

Traumatic-experience-related characteristics include perception of trauma. Horowitz et al. [23] developed a method for measuring a breast cancer patient’s degree of trauma perception. The Korean version of the Impact of Event Scale-Revised (IES-R-K) [24] was used, though some of the phrases were modified to suit the situation of the study subjects. The tool comprises 22 items and measures invasive, avoidance, and hyperarousal symptoms. The subjective symptoms experienced over the past month in relation to special trauma events were evaluated by individuals. The measurement uses a 5-point Likert scale, ranging from 0 (“not at all”) to 4 points (“there is often”). The scores range from 0 to 88, with higher scores indicating higher severity of symptoms. 

### 2.4. Statistical Analysis

Data were analyzed using IBM SPSS 23.0 software, and the effectiveness of the PTG program was evaluated. The general characteristics of the participants were evaluated using descriptive statistics (frequency, percentage, mean, standard deviation). Paired t-tests were used to compare outcomes before the program, immediately after the end of the program, and one month after the end of the program.

## 3. Results

The results indicated that PTG increased immediately after program completion (T2) (72.87/105 points) and one month later (T3) (72.23/105 points), as compared to the baseline (60.66/105 points). Furthermore, statistically significant changes in PTG occurred immediately after the program and one month later (*p* < 0.001). More specifically, all PTG subdomains (related to others, new possibilities, personal strength, spiritual changes, and appreciation of life) significantly differed immediately after the program ended and one month later, as compared to the baseline (Table 2).

Intrusive rumination decreased immediately after the program (10.66/30 points) and one month later (11.46/30 points), as compared to the baseline (12.03/30 points). However, there were no statistically significant changes in intrusive rumination after the program (*p* = 0.107, *p* = 0.936). Deliberate rumination increased immediately after program completion (20.24/30 points) and one month later (19.23/30 points), as compared to the baseline (18.53/30 points). However, statistically significant changes in deliberate rumination only occurred immediately after the program (*p* = 0.038) (Table 2).

Among aspects of distress, anxiety decreased immediately after the program (6.34/21 points) and one month later (5.74/21 points), as compared to the baseline (7.13/21 points). There were also statistically significant changes in anxiety immediately after the program (*p =* 0.038). Similarly, depression decreased immediately after the program (6.63/21 points) and one month later (6.86/21 points), as compared to the baseline (7.34/21 points). However, there were no statistically significant changes in depression immediately after the program (*p =* 0.104) or one month later (*p =* 0.517) (Table 2).

Traumatic perception decreased immediately after the program (38.95/88 points) and one month later (39.03/88 points), compared to the baseline (42.74/88 points). There were also statistically significant changes in traumatic perception immediately after the program (*p* = 0.081) and one month later (*p =* 0.059) (Table 2).

## 4. Discussion

This study confirmed the effectiveness of a PTG program developed to support young women diagnosed with breast cancer in recovering and returning to daily life, including work. Most patients with breast cancer have traumatic experiences during their diagnosis and treatment. Therefore, this study used the PTG model [4] to develop a program to increase PTG by reducing intrusive rumination and increasing deliberate rumination through including self-disclosure and social support resources.

Previous studies have shown that PTG-related interventions include meaning-based therapy [25,26], acceptance and commitment therapy [27,28], mindfulness and meditation [29,30], and writing [31,32]. Most of the cognitive and psychological interventions currently provided have not been developed based on theoretical models related to PTG. Therefore, simply enhancing PTG or reducing psychological difficulties such as distress may not be effective. In the future, programs should be developed based on theories of PTG. Furthermore, the mechanism through which this effect promotes PTG should be examined.

The contents of the program were constructed by identifying the needs of patients with breast cancer. A previous study indicated that it is necessary for patients to eat and exercise, manage a changed appearance, alleviate difficulties when balancing work and treatment, and share feelings with like-minded individuals [33]. Therefore, our program included education about understanding the meaning of breast cancer, planning a new life after cancer, coping with post-cancer situations, and living daily life regarding cancer treatment. Participants in the program made active efforts to find a new meaning of life despite practical difficulties. This approach differs from other PTG interventions, as it does not involve short-term coping with trauma or cognitive avoidance. For young breast cancer patients wanting to return to daily life, active efforts to advance into PTG may be viewed as a problem-solving or coping mechanism. This perception suggests that cognitive and behavioral coping mechanisms must be combined to experience the positive results of PTG [34]. According to the PTG model, providing education for coping with active problem-solving behavior after cancer diagnosis and treatment may result in higher levels of PTG when converted into actual behavior; this trend is a positive indicator of adaptation [35]. When patients with cancer experience high levels of PTG, they can attain active lives and positive life outlooks. When this state is achieved, more health-promoting activities can be undertaken, which further helps breast cancer patients return to work and maintain healthy bodies and minds.

Among participants of this study, the mean PTG score improved immediately after the program ended and one month afterward; furthermore, this difference was statistically significant (*p* > 0.001). Additionally, the mean scores improved for total PTG and all its five subdomains immediately after the intervention and one month afterward. These results confirm that the program was effective in enhancing PTG. In a previous study, Danhauer et al. [12] examined the PTG of patients diagnosed with breast cancer at six-month intervals and found a slight improvement in the mean scores. In contrast, the average scores in this study increased by about 12 points in only one month, suggesting that the program is highly effective in improving PTG. Additionally, self-disclosure based on the PTG model decreased intrusive rumination, and increasing the intentional rumination about medical teams and self-help group supports led to increased PTG. However, the PTG score tended to decrease slightly from the end of the intervention to one month later. It may be natural for the effect of the intervention to decrease after its completion. Additional simple educational materials or videos may be required, depending on the degree of PTG reduction.

This program was conducted in one of the largest hospitals in Korea. Due to the nature of medical care in Korea, most patients visit from far away areas. Thus, participants had to visit the hospital regularly (four times a month). Recruiting participants among younger breast cancer patients (under 50) was especially difficult. The program’s effectiveness had to be confirmed through a pre-test/post-test study, with one group and no control condition. Therefore, we could not observe a spontaneous increase in PTG over time, the most significant limitation in evaluating the results’ validity. In the future, the program’s effectiveness should be confirmed through a larger experiment–control group study. Furthermore, repeated studies are needed to confirm the program’s long-term effects. Finally, since this study targeted a specific ethnic minority in Asia, programs should be developed and studied to examine the effects concerning cultural differences and suitability for various races/ethnicities.

The methods used in this study can be immediately applied to other clinical settings, as the program was operated by a nurse in a hospital environment. Furthermore, as the program did not yield any dropouts, its clinical applicability was confirmed. Many PTG facilitation programs focus only on psychological approaches, such as cognitive behavioral therapy. However, this study will be helpful for promoting patients’ growth by strengthening healthy behaviors and coping mechanisms through multidisciplinary education. Additionally, this program can help patients prepare for returning to daily life.

## 5. Conclusions

This study confirmed the positive effect of a PTG program for young breast cancer patients based on the PTG model. Patients who participated in the program had significantly higher PTG scores after the intervention, indicating its effectiveness. Additionally, the rates of deliberate rumination among patients increased, while the symptoms of anxiety decreased. This program can help breast cancer patients return to their daily lives with a healthy body and mind. It may also be used to help prepare patients for returning to work. As the program was developed and operated by nurses and confirmed to be effective, it may be easily and widely applied to patients in clinical practice. In the future, controlled studies with large samples should confirm the effectiveness of the program. In addition, repeated studies and measures are needed to determine its long-term effects.

## Figures and Tables

**Table 1:** General characteristics of this study (n = 38).

Characteristics	n (%)	Mean ± Standard Deviation
Age (years)		42.21 ± 4.84
30s	12 (31.6)	
40s	26 (68.4)	
Spouse presence, yes	30 (78.9)	
Religion, yes	26 (68.4)	
Education, ≥university	36 (94.8)	
Monthly household income		
<USD 3000	5 (13.2)	
≥USD 3000	33 (86.8)	
Child existence, yes	30 (78.9)	
Cancer stage		
I	13 (34.2)	
II	12 (31.6)	
III	12 (31.6)	
IV	1 (2.6)	
Time since diagnosis (months)		13.84 ± 16.27
<6	7 (18.4)	
6–12	21 (55.3)	
>12	10 (26.3)	
Operation, yes	37 (97.4)	
Total mastectomy	15 (39.5)	
Breast conserving surgery	22 (57.9)	
Chemotherapy, yes	29 (76.3)	
Radiation therapy, yes	28 (73.7)	
Anti-hormone therapy, yes	21 (55.3)	
Occupation type		
Managers	5 (13.2)	
Professionals and related workers	11 (28.9)	
Clerks	21 (55.3)	
Sales workers	1 (2.6)	
Work period (months)		127.29 ± 90.15
Current occupational status		
Continued working	8 (21.1)	
Leave of absence	26 (68.4)	
Stopped working	4 (10.5)	

**Table 2:** Comparison of the effect of post-traumatic growth program.

Variables	Pre	Post	One Month Later	Pre/Post *p*	Pre/One Month Later *p*
	Mean ± SD	Mean ± SD	Mean ± SD		
Post-traumatic growth					
Total	60.66 ± 19.09	72.87 ± 16.00	72.23 ± 17.96	<0.001	<0.001
Relating to others	19.61 ± 7.08	23.58 ± 6.21	23.83 ± 6.57	<0.001	<0.001
New possibilities	14.18 ± 4.87	17.11 ± 4.08	16.91 ± 4.43	<0.001	<0.001
Personal strengths	11.61 ± 4.05	14.26 ± 3.37	14.00 ± 3.87	<0.001	0.001
Spiritual changes	4.74 ± 3.02	5.95 ± 2.71	5.94 ± 3.03	0.001	0.003
Appreciation of life	10.53 ± 3.21	11.97 ± 2.38	11.54 ± 2.59	0.001	0.001
Rumination					
Intrusive rumination	12.03 ± 5.99	10.66 ± 5.10	11.46 ± 6.77	0.107	0.936
Deliberate rumination	18.53 ± 5.23	20.24 ± 3.81	19.23 ± 5.12	0.038	0.373
Distress					
Anxiety	7.13 ± 3.06	6.34 ± 3.15	5.74 ± 3.14	0.035	0.005
Depression	7.34 ± 3.19	6.63 ± 3.30	6.86 ± 3.67	0.104	0.517
Traumatic experience					
Trauma perception	42.74 ± 13.49	38.95 ± 13.42	39.03 ± 17.72	0.081	0.059

## Data Availability

Data are available upon request.
